# Supraspinatus tendon reconstruction using open infraspinatus tendon shift and autologous biceps tendon interposition grafts

**DOI:** 10.1007/s00402-021-03757-8

**Published:** 2021-01-23

**Authors:** Jörg Panzert, Pierre Hepp, Mareike Hellfritzsch, Almut Sasse, Jan Theopold

**Affiliations:** 1 Orthopädiezentrum Sachsenortho, Breitscheidstrasse 13, 08209 Auerbach, Germany; 2 Department of Orthopedics, Trauma and Plastic Surgery, University of Leipzig, Liebigstrasse 20, 04103 Leipzig, Germany

**Keywords:** Rotator cuff tear, Biceps tendon transfer, Supraspinatus tendon, Infraspinatus shift, Defect arthropathy, Reconstruction technique, Massive tear

## Abstract

**Introduction:**

Inferior tendon quality, wide retraction, and tendon stumps that cannot be mobilized define the limits of what is technically feasible for open and arthroscopic rotator cuff reconstruction. The aim of this study was to develop a procedure that enables the open reconstruction of otherwise non-reconstructable rotator cuff tears.

**Methods:**

From 2014 to 2018, 23 operations were performed on 21 patients (mean age 63) using open procedure involving separating the infraspinatus tendon from the point of insertion on the greater tubercle and mobilizing it proximally and ventrally into the defective area. Any remaining defects were augmented using an autologous biceps tendon interposition graft. This augmentation was performed to achieve complete closure of the defect. Furthermore, the augmentation of the rupture zone was intended to strengthen the tendon stumps of the SSP and ISP to better neutralize the initial tensile forces. After a postoperative period of 12 months–4 years, clinical examination and functional tests were carried out, the Constant score was determined, and radiological and magnetic resonance imaging check-up examinations were performed.

**Results:**

The technique resulted in a low-tension closure of an otherwise “non-repairable” superior rotator cuff defect. All patients experienced a significant functional improvement, a reduction in pain, and an increase in muscle strength. An improvement in the Constant score from 48 points preoperatively to 87 points postoperatively (*p* < 0.05) was observed. In 19 patients (90%), the magnetic resonance image showed an intact reconstruction. Re-rupture was seen in three patients (14%).

**Conclusion:**

The surgical procedure using infraspinatus tendon shift and autologous biceps tendon interposition grafts resulted in the successful reconstruction of otherwise non-reconstructable massive rotator cuff lesions. The complete closure of the defect was observed.

## Introduction

Non-reconstructable retracted supraspinatus (SSP) tendon defects pose a therapeutic challenge. Inferior tendon quality, wide retraction, and tendon stumps that cannot be mobilized define the limits of what is technically feasible for open and arthroscopic rotator cuff reconstruction. If superior rotator cuff lesions are not reconstructed, progressive cranialization of the humeral head can often occur, in addition to impaired function and strength. The decentering of the humeral head leads to pain and increasing functional restrictions in the shoulder, progressing right up to cuff arthropathy.

Depending on the severity of the tear, various treatment options are used to improve function and avoid irreversible long-term damage. These techniques include debridement with or without tenotomy of the long biceps tendon (LBT) [1], partial reconstruction with and without patch techniques, [2] superior capsule reconstruction [3] or absorbable subacromial spacers [4], tendon transfer methods [5, 6], and, as a last resort, the inverse shoulder prosthesis [7].

So-called local reconstruction techniques have been established in open shoulder surgery [8], but have played an apparently subordinate role since the advent of reconstructive arthroscopic shoulder surgery. Reconstruction techniques offer possibilities of extensive tendon mobilization in combination with augmentation procedures. Infraspinatus (ISP) transposition plays a crucial role in the reconstruction of superior, non-repairable lesions. The tendon is separated from the point of insertion at the greater tubercle and shifted proximally and ventrally into the defect area [8–10].

In contrast to tendon transfer methods, which aim at dynamic re-centering and shoulder joint function improvement, augmentation procedures reinforce static reconstruction. As a biological autologous interposition graft, the LBT has been identified as an augmentation option for massive rotator cuff tears and has received considerable attention [11]. As a rule, the LBT is tenotomized near the point of insertion and is distally configured as augmentation material for non-repairable tears [12–16]. “Overall, 8 (38%) traumatic ruptures and 15 (62%) chronic injuries of the rotator cuff were found. In 23 patients, the supraspinatus muscle was retracted to the glenoid level. In 8 (38%) cases, a rupture size of 3–5 cm was found. In 15 (62%) cases, a global tear with no residual tendon was found.

Local ISP transposition and additive augmentation with the LBT is a combination procedure consisting of local dynamic transfer of a tendon, with an authentic external rotation function, and identical innervation with autologous locally available augmentation material. This procedure appears to be a good alternative to non-local tendon transfer methods and allogenic augmentation methods in the case of a completely or partially preserved ISP tendon. Currently, there are largely no clinical studies presenting this method in literature. Results of a modification in an ISP/teres minor transfer in combination with autologous biceps tendon augmentation showed promising functional results after an average of 12 months [17].

The purpose of this study was to develop a procedure which enables the reconstruction of otherwise non-reconstructable rotator cuff tears.

## Methods

Twenty-three operations were performed on 21 consecutive patients (13 men (62%) and 8 women (38%)) using the technique presented here. The average age at the time of surgery was 63 (range 48–76) years. Overall, 8 (38%) traumatic ruptures and 15 (62%) chronic injuries of the rotator cuff were found. In 23 patients, the supraspinatus muscle was retracted to the glenoid level. In 8 (38%) cases, a rupture size of 3–5 cm was found. In 15 (62%) cases, a global tear with no residual tendon was found. All patients met the inclusion and exclusion criteria (Table 1). After a post-surgery period of 12 months to 4 years (average 22 (range 12–50) months), clinical examination and functional tests were carried out, the Constant score was determined, and radiological and magnetic resonance imaging (MRI) check-up examinations were performed. The present study was performed retrospectively, and patients were treated between 2014 and February 2018.

**Table 1:** Inclusion and exclusion criteria

Inclusion	Exclusion
Age > 18 years	Pregnancy
Non-reconstructable rotator cuff tear	Accompanied neurovascular injuries
Signed informed consent	Lack of consent to participate
Moderate load resistance training	

### Surgical technique

**Fig. 1:**
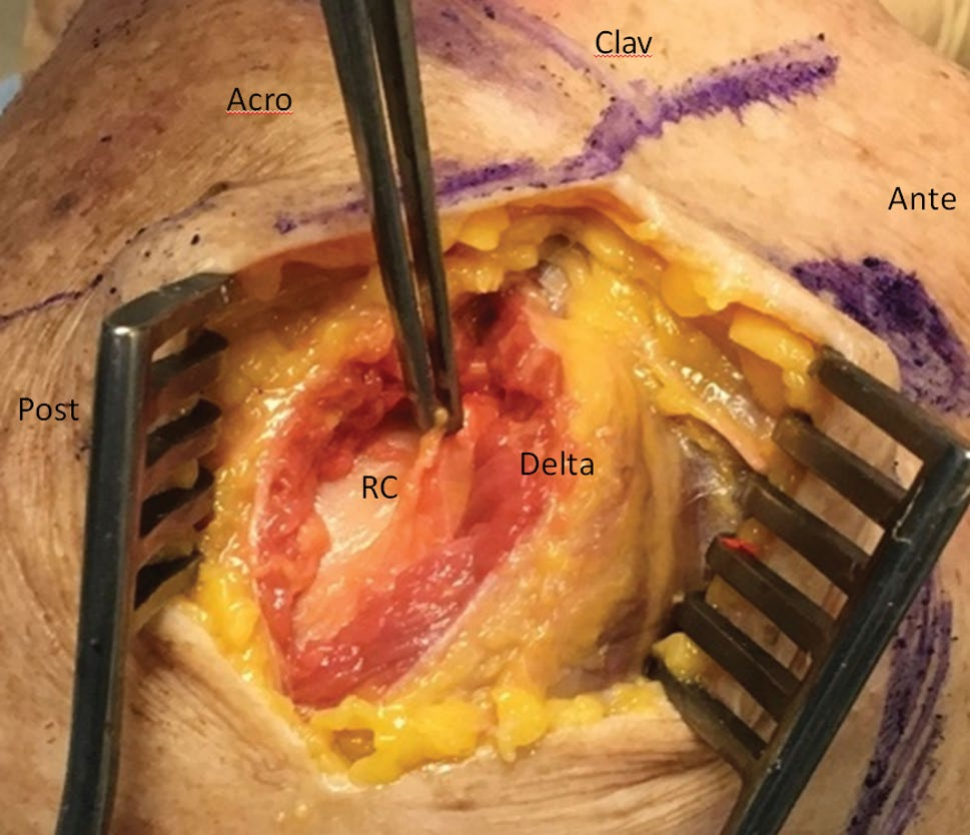
Skin incision and access. Skin incision in extension of the front edge of the acromion with subsequent splitting of the deltoid muscle. The fibers are cut in the direction of the fibers. (*Acro* acromion; *RC* rotator cuff; *Delta* deltoid muscle; *Ante* anterior; *Post* posterior)

With the patient in the “beach chair” position, an approximately 3 cm long skin incision was made to extend the front edge of the acromion, of which 1–2 cm runs from above the lateral acromion (Fig. 1). The deltoid muscle was then split. The fibers were severed and the fiber strands were detached from the lateral front edge of the acromion. A scalpel was then used to dissect a periosteal flap on the acromion.

This was followed by an acromioplasty with resection of the coracoacromial ligament. Here, the front edge of the acromion was osteotomized with a 1.5-cm-wide chisel, only to such an extent that the surface and thus the length of the acromion was retained. This avoids subsequent shortening of the acromial lever arm of the deltoid muscle. The direction of the osteotomy was aimed at the “dome” of the lower surface of the acromion. The osteotomy wedge had a bottom edge length of approximately 1.5 cm and covered the entire width of the acromion up to the acromioclavicular (AC) joint. Osteophytes on the lower edge of the clavicle were ablated. Finally, the subacromial space was smoothed with a file.

**Fig. 2:**
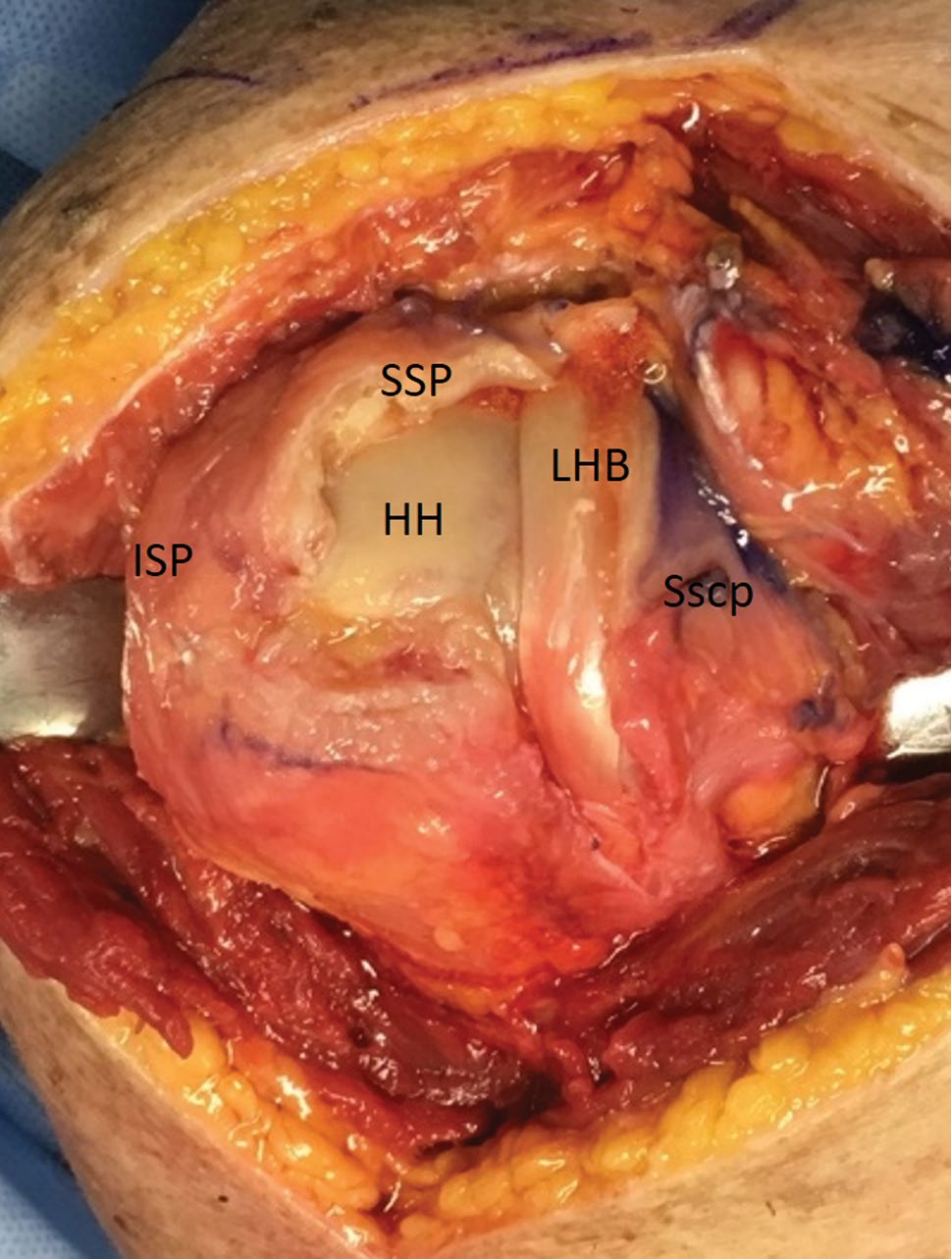
Tear configuration and reduction. On the anatomical model, two non-reconstructable tendon injuries in the region of the supraspinatus (SSP) were visible in remnants below the acromion. The infraspinatus tendon (ISP) still inserts at the footprint. (*LHB* unstable long biceps tendon; *Sscp* subscapularis tendon; *HH* humeral head)

Manual digital mobilization removed adhesions around the entire humeral head between the deltoid and the rotator cuff. The rotator cuff was then inspected and the tear pathology analyzed (Fig. 2).

**Fig. 3:**
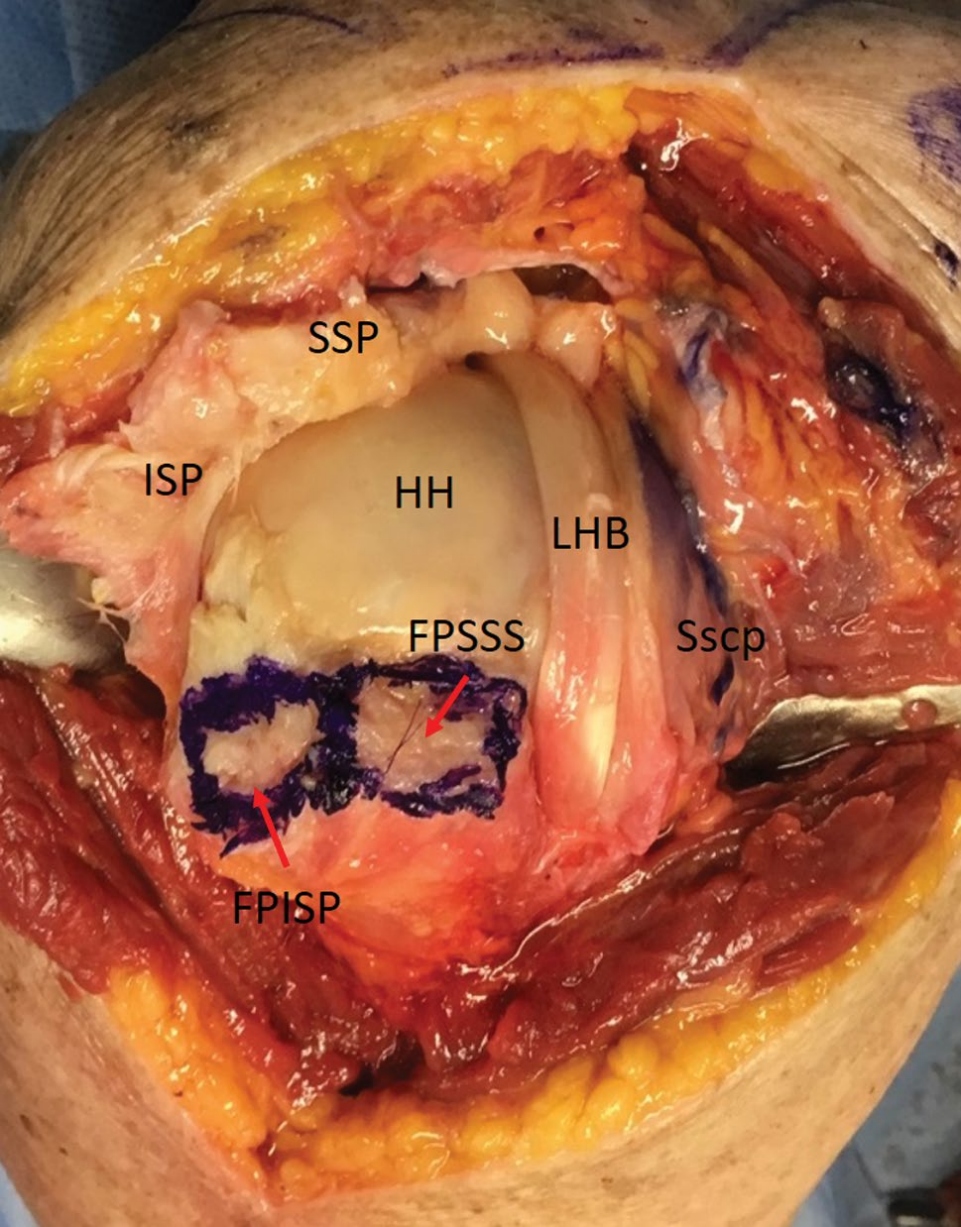
Footprint of the tendons. Large-scale detached ISP with representation of the footprint (*FPISP* footprint infraspinatus; *FPSSP* footprint supraspinatus) and the dorsal capsule. (*LHB* unstable long biceps tendon; *Sscp* subscapularis tendon; *HH* humeral head)

The residual SSP was now mobilized on the joint and acromial side. Using a scalpel, the ISP was detached from the greater tubercle starting at the myotendinous junction. As a rule, the layer between the joint capsule and the ISP muscle can be found easily here. In this layer, the capsule was dissected in the direction of the tendon insertion and the ISP tendon was severed from the point of insertion on the greater tubercle (Fig. 3). The nearly complete detachment of the ISP with the dorsal capsule can be seen from a clear gain in elasticity. The posterior part of the Tendon is still attached to the Bone. So, there will not be any defect in the posterior rotator cuff. This was followed by removing exophytes with a luer and freshening the entire cartilage–bone junction on the greater tubercle. From the anatomically determined tent-like point of insertion of the SSP and ISP, a curved, circular point of insertion was created, with visible minor bleeding.

**Fig. 4:**
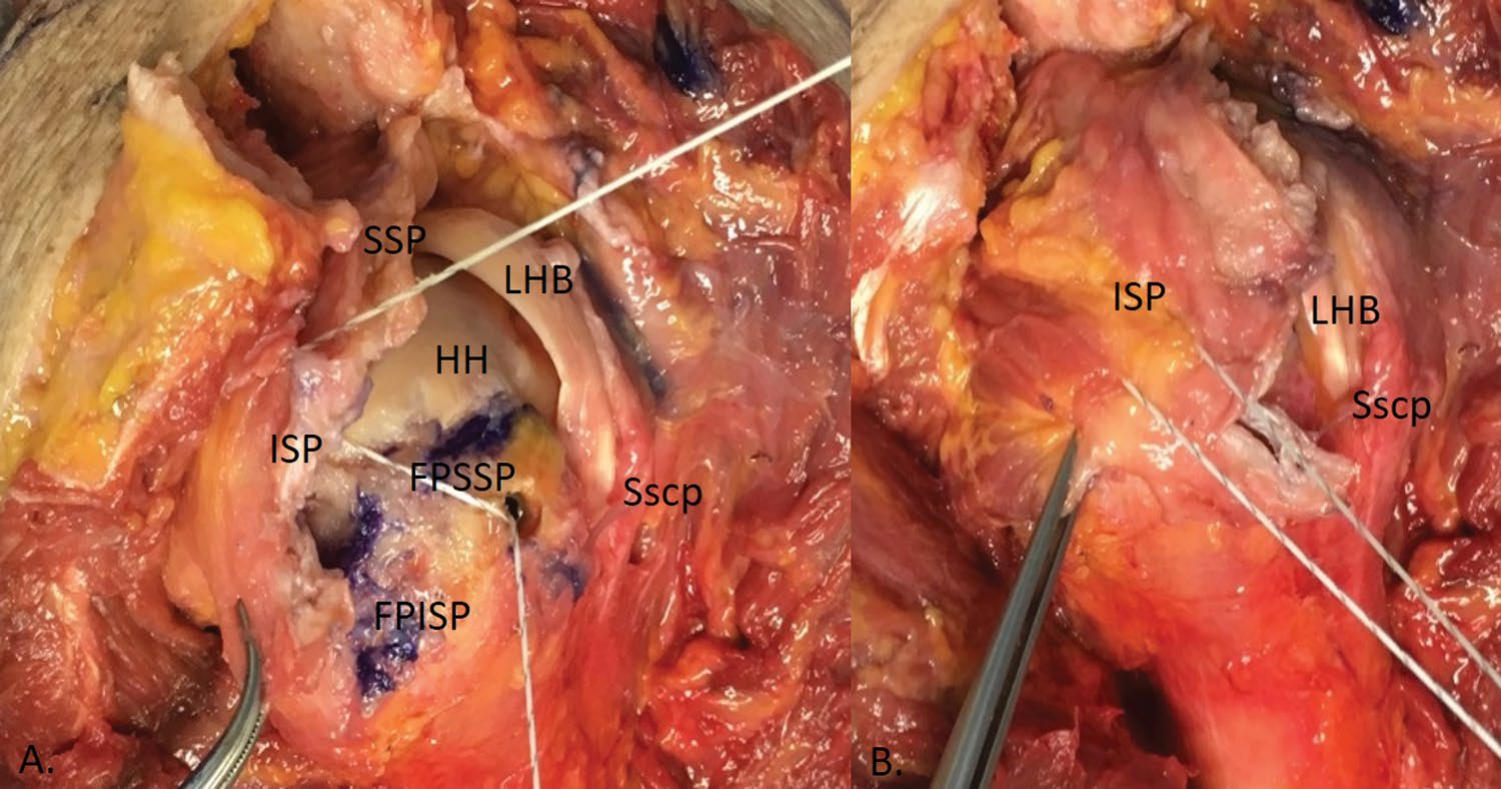
Placement of the suture anchors and trial reduction. **a** Before reduction, introduction of the first suture anchor to re-fix the mobilised infraspinatus tendon (ISP) with a screw anchor. **b** After reduction of the infraspinatus tendon (ISP), there is still a defect in the anterior region of the supraspinatus (SSP). However, in almost all cases, the defect is reduced to such an extent that a complete reconstruction can be achieved with the help of the proximal long biceps tendon. (*FPISP* footprint infraspinatus; *FPSSP* footprint supraspinatus; *LHB* unstable long biceps tendon; *Sscp* subscapularis tendon; *HH* humeral head)

After extensive preparation, a suture anchor was inserted to refix the tendon of the ISP muscle that had now been displaced ventrally. In almost all cases, the defect was significantly reduced. In the case of a remaining defect in the area of the interval and if SSP was still present, interposition was performed medially in the area of the anterior margin to achieve gain at this point. The course of the biceps tendon is then parallel to the original course of the biceps tendon (Fig. 6a). If a defect remained laterally, the biceps tendon was stitched dorsally over the area of the footprint 90° to the defect or to the tendon stump for reinforcement. (Fig. 6b). This was provided so that a complete reconstruction could be achieved with the aid of the proximal LBT (Fig. 4).

**Fig. 5:**
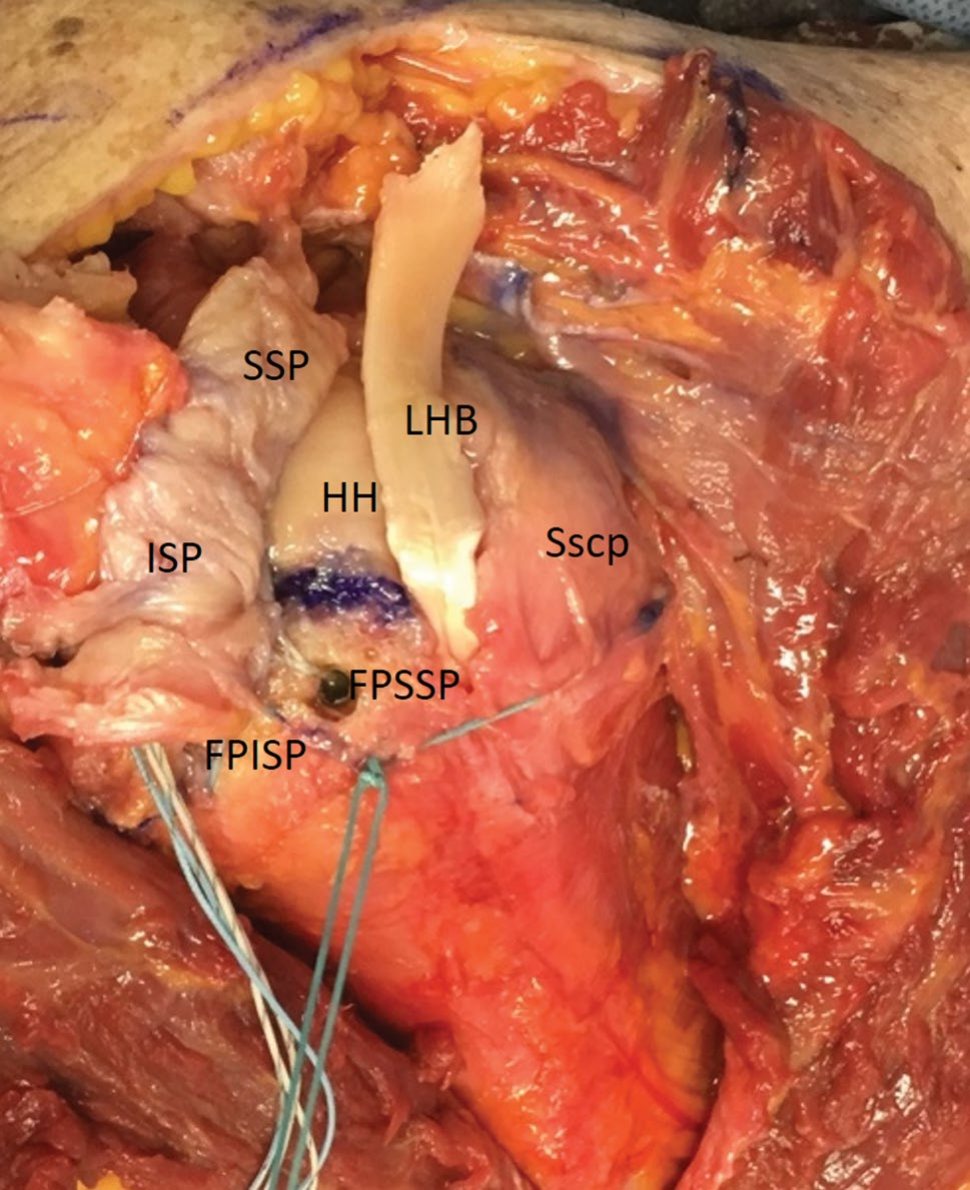
Tenodesis of the long biceps tendon (LHB). The transosseous tenodesis of the long biceps tendon (LHB) in the region of the bicipital groove. A second anchor to secure the tendon dorsally has already been inserted. The ends of the tendon that are still free have been flipped dorsally for better clarity at the anatomical specimen. (*ISP* infraspinatus tendon; *SSP* supraspinatus tendon; *FPISP* footprint infraspinatus; *FPSSP* footprint supraspinatus; *Sscp* subscapularis tendon; *HH* humeral head)

The tenodesis of the LBT was performed as a soft tissue tenodesis in the area of the proximal bicipital groove, either transosseously or with a suture anchor (Fig. 5).

**Fig. 6:**
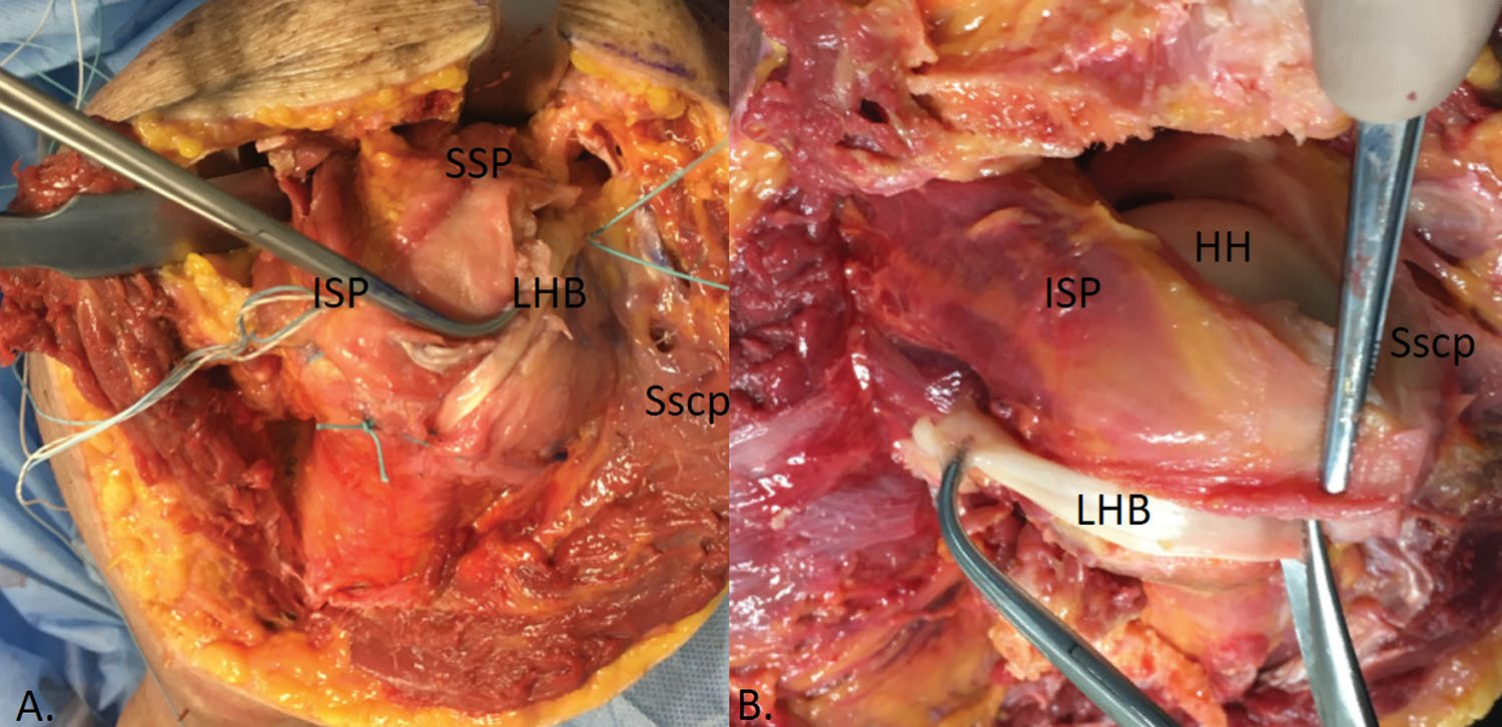
Positioning of the long biceps tendon (LHB). Interposition of the long biceps tendon (LHB) in accordance with the circumstances of the defect. It is either sutured medially with the retracted tendon stump of the SSP or turned 90° in the dorsal direction over the greater tubercle. (*ISP* infraspinatus tendon; *SSP* supraspinatus tendon; *Sscp* subscapularis tendon)

The LBT was interposed in accordance with the circumstances of the defect. It was either sutured medially with the retracted tendon stump of the SSP or turned 90° in the dorsal direction over the greater tubercle (Fig. 6).

**Fig. 7:**
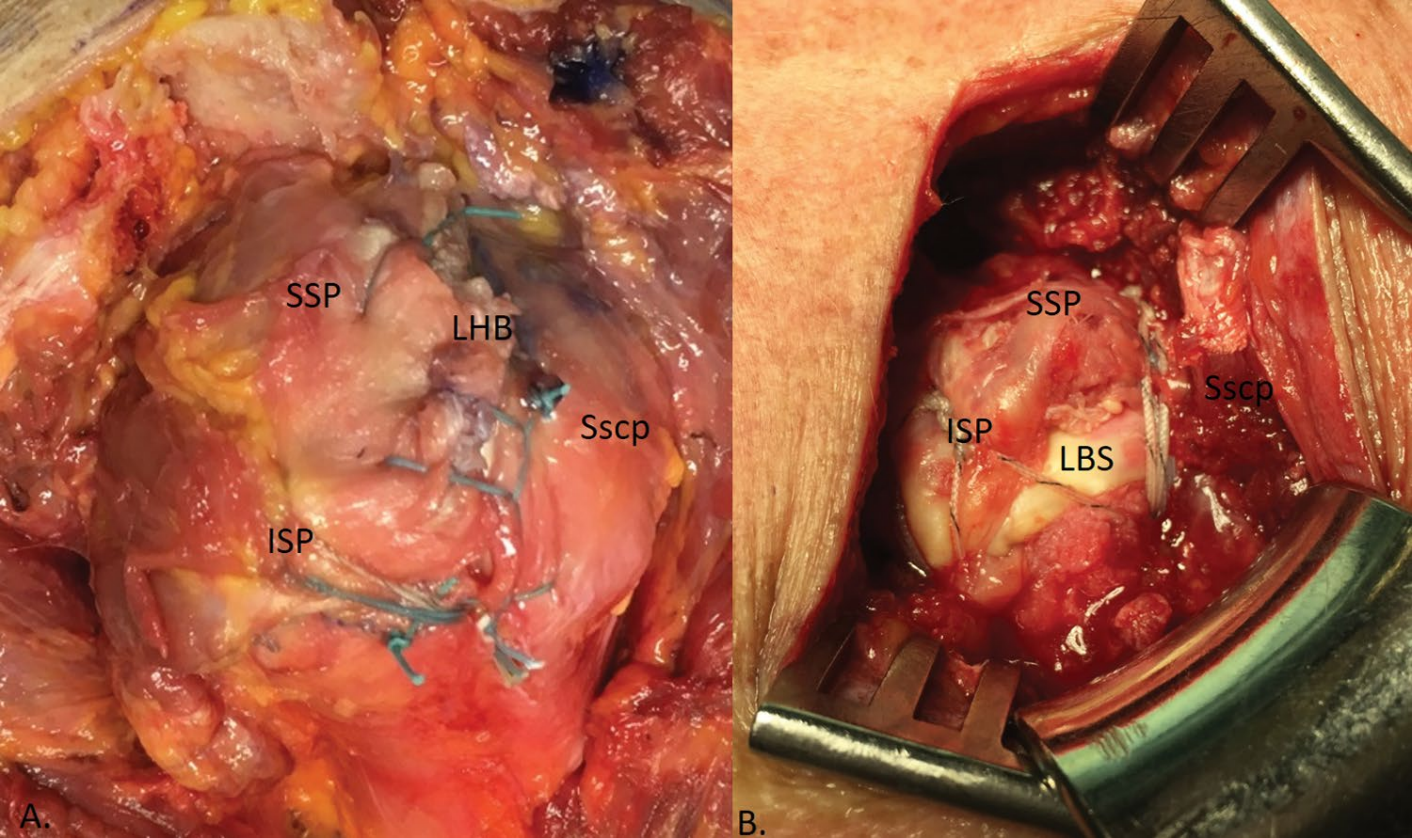
Cuff after the complete closure. Both possible variants after the cuff has been completely closed. **a** On the anatomical specimen, the long biceps tendon (LBT) was sewn into the defect of the SSP. **b** Intraoperatively, the LBT was sewn into the defect of the tendon dorsally, onto the footprint. (*ISP* infraspinatus tendon; *SSP* supraspinatus tendon; *Sscp* subscapularis tendon)

Both variants resulted in the cuff being completely closed (Fig. 7).

Finally, the wound was closed layer-by-layer and the skin suture was put in place.

### Postoperative care

The patients were immobilized with a shoulder abduction splint for four weeks and, starting from the third postoperative day, they were made to exercise with limited passive movements using a continuous passive motion (CPM) splint, combined with passive movement exercises guided by a physiotherapist. In the third postoperative week, active-assisted movements were carried out, up to as much as 70° abduction. After a follow-up ultrasound, the patients were gradually weaned off the orthosis from the fourth postoperative week, and this was followed by outpatient or inpatient rehabilitation.

### MRI evaluation

Preoperatively, 23 MRIs were performed and interpreted for this study by two independent shoulder surgeons. The tear configuration according to Bateman, the retraction of SSP according to Patte and the degree of fatness of SSP according to Goutalliere were evaluated and documented. At the time of follow-up, 23 control MRIs of the shoulders could be evaluated. The images were evaluated by two independent shoulder surgeons. The continuity of the reconstructed tendons and the degree of fatty degeneration of SSP according to Goutallier were assessed. The assessment of the fatty infiltration of the other muscles of the rotator cuff, especially the infraspinatus, in the MRI has not been performed.

### Statistical analysis

Results are reported as mean values and range. The Mann–Whitney test was used to compare pre- and postoperative data. The level of significance was set to *p* < 0.05. The statistical analysis was performed using SPSS (IBM, Version 24).

## Results

A total of 21 patients with 23 treated ISP displacements and autologous proximal biceps tendon augmentation were included. Table 2 summarizes the preoperative and postoperative epidemiological and clinical data of the patients.

**Table 2:** Summary of epidemiological and clinical data

Patient information (*n* = 23)		
Dominant side	Left/Right 0/23	
Cause	Trauma 8 (38%)	Degenerative 15 (62%)
Clinical results		
Constant Murley score	Preoperative	Postoperative
Total	48 points (min 24; max 73)	87 points (min 61; max 100)
Sub-groups		
Pain (0–15 points)	10 points (min 4; max 13)	2 points (min 0; max 8)
Abduction function	140° (min 15; max 180)	155° (min 90; max 180)
Anteversion function	145° (min 45; max 180)	158° (min 90; max 180)
External rotation function	55° (min 0; max 90)	90° (min 90; max 90)
Strength	3 points (min 2; max 5)	4 points (min 3; max 5)
Radiological results		
Verifiable re-rupture	3 (14%)	
	Preoperative	Postoperative
Retraction according to Patte:	23 × III°	
Tear size according to Bateman	8 × III°; 15 × IV°	
Goutallier SSP:	1° (min 0; max 2)	2° (min 1; max 4)
AH distance:	9 mm (min 6; max 11)	10 mm (min 7; max 13)
Decentering in accordance with Hamada:	min 0; max 7 mm	min 0; max 7 mm

All patients experienced significant improvement in shoulder function with an increased range of motion, a reduction in pain, and increased strength. This can be seen in the significant (*p* < 0.05) improvement in the Constant score from 48 points preoperatively to 87 points postoperatively. Overall, a substantial improvement of the initially limited external rotation of 45° was found.

In 19 patients (90%), the MRI scan showed an intact reconstruction. Three patients (14%) had a re-rupture. Despite intact reconstruction, the degree of fatty degeneration based on Goutallier increased from 1.1 preoperatively to 2.1 postoperatively in the region of the SSP muscle. The sutured tendon was morphologically indistinguishable from the refixed tendon, a defect in this area was not visible. A discontinuity could not be detected in the follow-up MRI.

No infections occurred in the postoperative course, but there was temporary axillary insufficiency (5%). Postoperative shoulder stiffness (10%) was seen in two cases but healed very well under non-surgical measures. One patient (5%) had to undergo surgical revision due to persistent pain. The performed MRI of this patient showed a partial defect of the SSP refixation. This defect was evaluated as a re-eruption of the cuff. During the re-operation, a persistent residual defect was found which could be completely closed during the procedure.

## Discussion

The most important finding of the present study was that surgery using infraspinatus tendon shift and autologous biceps tendon interposition grafts resulted in successful reconstruction of otherwise non-reconstructable massive rotator cuff lesions. Massive lesions constitute a high proportion of rotator cuff injuries. This is mainly due to the spontaneous degenerative tears that occur in old age, delayed medical consultations without a timely diagnosis, tolerable complaints in the case of minor traumas, and a relatively high level of acceptance of functional restrictions of the shoulder in many patients. While asymptomatic injuries should preferably be treated non-surgically, symptomatic massive lesions that come with a level of suffering have to be treated quickly with surgery, because the size of the tears, degree of retraction, and pain symptoms usually increase with time [18]. A large number of techniques for reconstructing the rotator cuff in case of massive lesions have, therefore, been developed in recent years. These range from debridement to tendon transfer techniques. So far, however, no surgical method has gained acceptance [19]. Partial reconstruction of the rotator cuff still represents the main surgical technique in use today [18, 20–22]. Partial repair is associated with a satisfactory functional outcome. However, complete reconstructions clearly show better Constant score values for strength, movement, and pain. Therefore, complete closure should also be attempted for massive lesions [23].

The surgical technique presented makes is possible to bridge the largest defect situations, even in the case of severely retracted (Patte III) massive lesions (Bateman IV). By nearly complete detaching the ISP from the greater tubercle and mobilizing it in the ventral direction, a reduction of 1–2 cm into the insertion area of the SSP is possible. The proximal part of the LBT above the bicipital groove often shows hypertrophy up to 1 cm wide in the case of superior lesions. With a length of up to 3 cm, it is possible to reduce the lesion by 3 cm^2^. Additionally, in contrast to treatment with tenotomy of the LBT alone, the functionality of the tendon is preserved. Compared to latissimus dorsi transfer, the technique is less invasive, and the often-difficult reprogramming from external rotation to abduction is eliminated, since the SSP and ISP insert at the greater tubercle with a common tendon plate.In the 12-month clinical follow-up, all patients with a mean Constant score of 90 points were subjectively very satisfied with the surgical result achieved. In respect to external rotation, we have seen an improvement of 50%. We explain this with the preservation of the dorsal structures ISP/Teres minor and the restored continuity of the rotator cuff. The functional benefits found here might be related to the complete closure of the rotator cuff on the one hand and to a re-centring of the humeral head. Furthermore, the improvement in pain could be due to the relief of the suprascapular nerve.

The results of other procedures, such as superior capsular repair (SCR) and subacromial spacers, are also similar to the reconstruction technique presented here. However, the technique described here appears to be advantageous, as it is a functional restoration of the cuff. Due to the restored muscular integrity of the cuff, it is a curative procedure, but does not fundamentally alter the anatomy as in a complete muscle transfer. In SCR and subacromial spacers, the re-centring improves mobility and reduces pain [24–27]. Also, the Constant score showed a 50% increase in subacromial spacers [27]. And also the SCR shows a marked improvement in the Constant score, at least in the 2–3 year follow-ups [24, 28] In the results presented here, as with SCR, an improvement in abduction and anteversion could be achieved [3, 25, 26, 29] For the external rotation, there are also comparable improvements to SCR [3].

An intact reconstruction was confirmed in the MRI scans of 85% of the patients operated on. The 90 points obtained in the Constant scores of the 17 patients with an intact reconstruction result are significantly higher than those obtained after partial reconstructions [30, 31]. MRI shows complete healing of the LHB tendon into the defect. In the MRI images, the tendon and can no longer be distinguished from the cuff tissue. The postoperative MRIs show re-rupture rates of 15% in the patient group examined. This result is comparable to the tear rates of 10–36% given in the literature for poorly retracted, easily reconstructable single-tendon lesions [18, 32]. The three patients (15%) with re-ruptures are subjectively satisfied, with low pain levels and good shoulder function. These can, therefore, be classified as asymptomatic re-lesions that do not require any further surgical treatment. With a Constant score of 80 points, these patients are above the 65 points of conservatively treated patients [17] and the 67 points of those after partial reconstruction [33]. This means that the Constant score values are higher than those of comparable surgical methods [12, 33–37].

The postoperative MRI images show an increase in the degree of fatty degeneration despite a largely intact rotator cuff. This is surprising, since the resumption of the muscle function of the SSP and ISP would most likely result in recovery and strengthening of the muscle.

### Limitations

The present study is limited by the small sample size and the absence of an appropriate control. Thus, the beneficial effects of using an ISP shift and the LBT should not be overestimated. Due to the fact that the study has not yet been completed, long-term control is still pending. Based on the current good results, however, a statement, at least regarding the results after one year, is possible. Larger clinical studies including a control group are needed to determine the advantages of using this technique. Due to the present results, a direct statement about the change in traction forces, with and without interposition of the LHB, is not possible. Biomechanical studies should be carried out for this purpose.

## Conclusion

The present study aimed to be the first experience with the combination of an ISP shift and the interposition of the LBT. The surgical method described here achieves very good clinical results in the case of massive rotator cuff lesions due to the complete closure of the defect.

## Abbreviations

AC: Acromioclavicular joint
CPM: Continuous passive motion
ISP: Infraspinatus
LBT: Long biceps tendon
MRI: Magnetic Resonance Imaging
SSP: Supraspinatus
